# Supplementary material for: miR614 Expression Enhances Breast Cancer Cell Motility
Source: Int J Mol Sci. 2020 Dec 24;22(1):112. doi: 10.3390/ijms22010112 (PMC7801944; doi:10.3390/ijms22010112)
Supplement: Supplementary file 1 [file ijms-22-00112-s001.zip › Supps resubmitted/ijms-1033685 (4).docx]

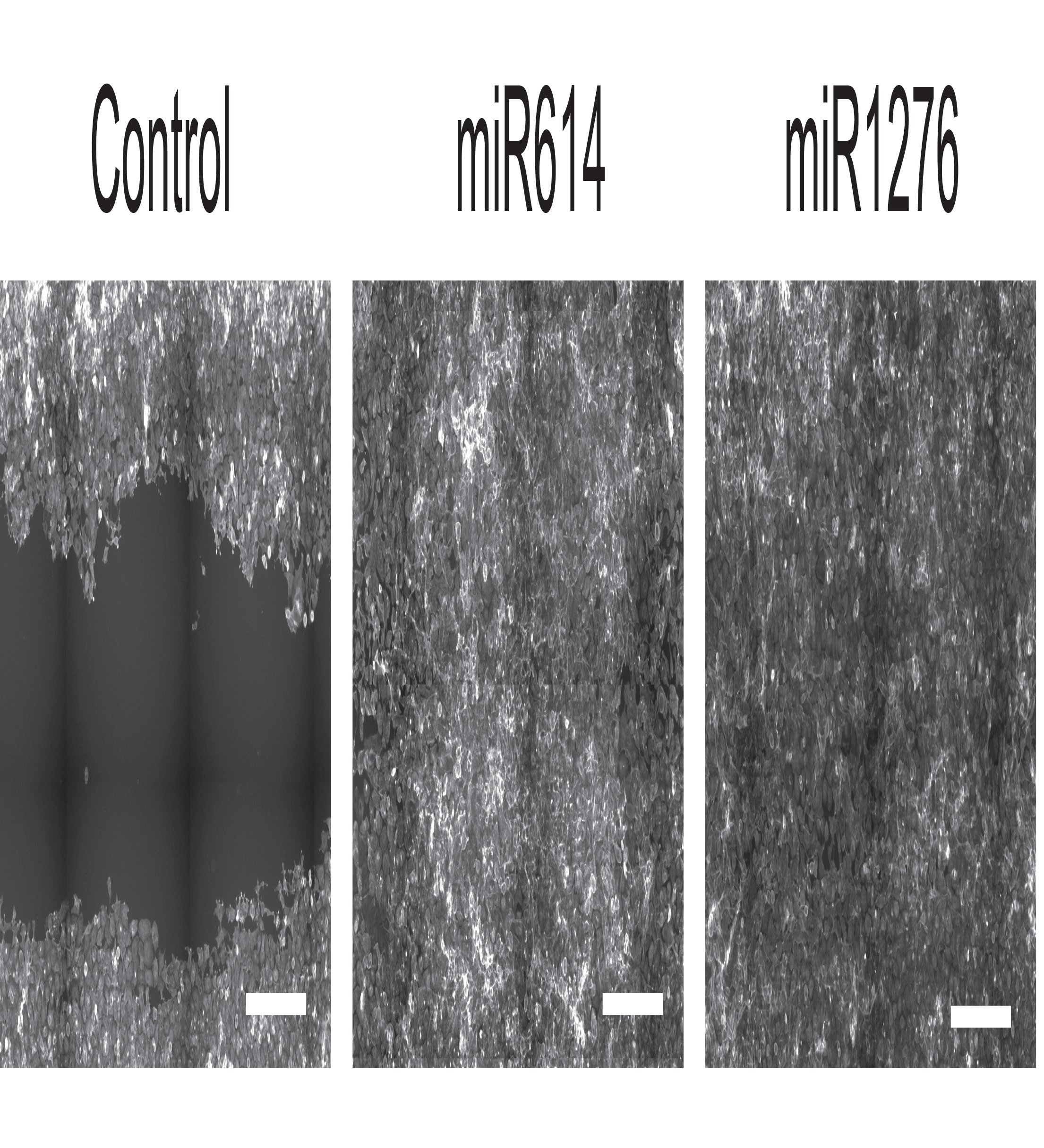


**Figure S1. Wound closure of MCFDCIS cells from a high content screen.** Representative raw data images of control, miR614 and miR1276 mimic transfected cells that were analyzed as part of a high content screen testing 879 miRNA mimics are shown. Cells were fixed and stained with phalloidin (white).

**
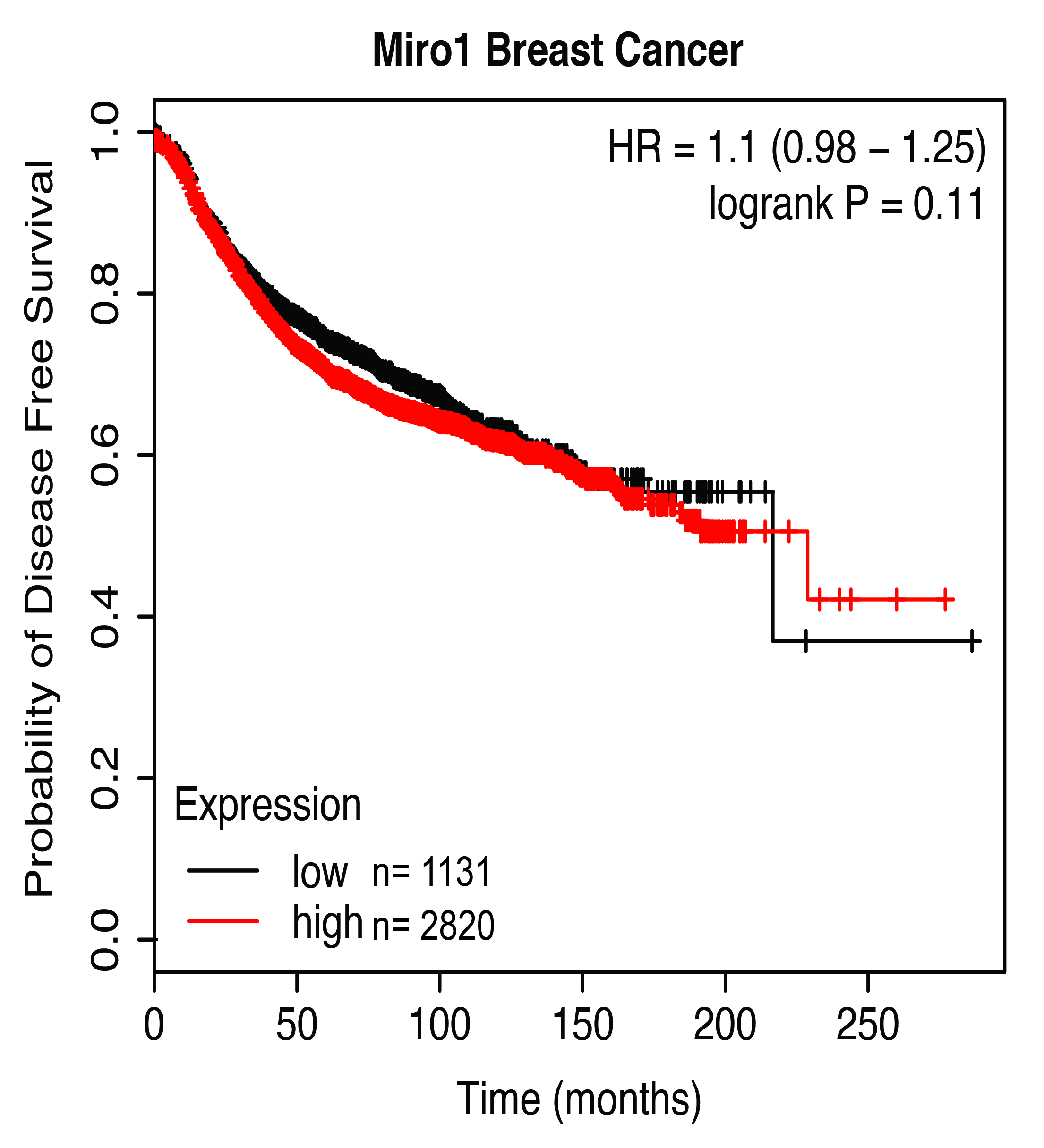
Figure S2. Analysis of the correlation between Miro1 expression and the outcome of breast cancer patients.** Kaplan-Meier curves showing the disease free survival of breast cancer patients classified as “Miro1 high” and “Miro1 low” based on Miro1 mRNA expression. Survival differences were compared by log-rank test.

**Movie S1. Control wound closure.** Time-lapse imaging of control MCFDCIS-H2B:GFP cells during wound closure. Bright-field and GFP (green) signal is shown.

**Movie S2. miR614 induced wound closure.** Time-lapse imaging of miR614 transfected MCFDCIS-H2B:GFP cells during wound closure. Bright-field and GFP (green) signal is shown.

**Movie S3.** **miR1276 induced wound closure.** Time-lapse imaging of miR1276 transfected MCFDCIS-H2B:GFP cells during wound closure. Bright-field and GFP (green) signal is shown.

**Movie S4. Control spontaneous motility.** Time-lapse imaging of control subconfluent MCFDCIS-H2B:GFP cells. Bright-field and GFP (green) signal is shown.

**Movie S5. miR614 induced spontaneous motility.** Time-lapse imaging of miR614 transfected subconfluent MCFDCIS-H2B:GFP cells. Bright-field and GFP (green) signal is shown.

**Movie S6. miR1276 induced spontaneous motility.** Time-lapse imaging of miR1276 transfected subconfluent MCFDCIS-H2B:GFP cells. Bright-field and GFP (green) signal is shown.

**Table S1. List of genes suppressed or induced by miR614 expression in MCFDCIS cells.** MCFDCIS cells were transfected with control or miR614 mimics for 48 h. Gene expression was determined using Illumina bead microarray. Genes that were suppressed or induced ≥2-fold in miR614 transfected cells compared to control cells are listed. Z-scores are shown.

**Table S2. List of genes suppressed or induced by miR614 expression in MDAMB231 cells.** MDAMB231 cells were transfected with control or miR614 mimics for 48 h. Gene expression was determined using Illumina bead microarray. Genes that were suppressed or induced ≥1.5-fold in miR614 transfected cells compared to control cells are listed. Z-scores are shown.

**Table S3. List of genes suppressed by miR614 in MCFDCIS and MDAMB231 cells.** The overlap if the mir614 suppressed gene lists from **Table S1** and **Table S2**.

**Table S4. List of antibodies used.**

**Table S5. List of miRNA mimics and siRNAs used.**

**Table S6. List of qPCR primers used.**
